# Tissue and salinity specific Na^+^/Cl^−^ cotransporter (NCC) orthologues involved in the adaptive osmoregulation of sea lamprey (*Petromyzon marinus*)

**DOI:** 10.1038/s41598-021-02125-1

**Published:** 2021-11-22

**Authors:** A. Barany, C. A. Shaughnessy, R. M. Pelis, J. Fuentes, J. M. Mancera, S. D. McCormick

**Affiliations:** 1 Department of Biology, Faculty of Marine and Environmental Sciences, Campus de Excelencia Internacional del Mar (CEI-MAR), University of Cádiz, Puerto Real, 11519 Cádiz, Spain; 2 Centre of Marine Sciences (CCMar), University of Algarve, Campus de Gambelas, 8005-139 Faro, Portugal; 3 Conte Anadromous Fish Research Laboratory, Eastern Ecological Science Center, U.S. Geological Survey, Turners Falls, MA 01376 USA; 4 Graduate Program in Organismic and Evolutionary Biology, University of Massachusetts, Amherst, MA 01003 USA; 5 Department of Pharmaceutical Sciences, Binghamton University, State University of New York, Johnson City, NY 13790 USA

**Keywords:** Molecular biology, Physiology

## Abstract

Two orthologues of the gene encoding the Na^+^-Cl^−^ cotransporter (NCC), termed *ncca* and *nccb*, were found in the sea lamprey genome. No gene encoding the Na^+^-K^+^-2Cl^−^ cotransporter 2 (*nkcc2*) was identified. In a phylogenetic comparison among other vertebrate NCC and NKCC sequences, the sea lamprey NCCs occupied basal positions within the NCC clades. In freshwater, *ncca* mRNA was found only in the gill and *nccb* only in the intestine, whereas both were found in the kidney. Intestinal *nccb* mRNA levels increased during late metamorphosis coincident with salinity tolerance. Acclimation to seawater increased *nccb* mRNA levels in the intestine and kidney. Electrophysiological analysis of intestinal tissue ex vivo showed this tissue was anion absorptive. After seawater acclimation, the proximal intestine became less anion absorptive, whereas the distal intestine remained unchanged. Luminal application of indapamide (an NCC inhibitor) resulted in 73% and 30% inhibition of short-circuit current (I_sc_) in the proximal and distal intestine, respectively. Luminal application of bumetanide (an NKCC inhibitor) did not affect intestinal I_sc_. Indapamide also inhibited intestinal water absorption. Our results indicate that NCCb is likely the key ion cotransport protein for ion uptake by the lamprey intestine that facilitates water absorption in seawater. As such, the preparatory increases in intestinal *nccb* mRNA levels during metamorphosis of sea lamprey are likely critical to development of whole animal salinity tolerance.

## Introduction

Lampreys are one of two surviving lineages of jawless fishes of the group Agnatha. Agnathans appear to have diverged during the late Ordovician period ~ 450 million years ago (mya)^1,2^ and persisted through several confirmed mass extinctions on Earth^3,4^. Adult lampreys have remained morphologically conserved based on a limited fossil record and have inhabited marine and estuarine environments from at least the late Devonian period ~ 360 mya^5^. Two episodes of whole-genome duplication likely occurred prior to the divergence of Agnatha^6^. Additional whole-genome duplications have been reported in the ancestors of modern salmonids^7^, castomids (suckers)^8^, and common carp^9^, and additional partial genome duplications have been reported elsewhere in fishes^10^. Given the basal phylogenetic position of Agnatha and the many genomic and phenotypic evolutionary transitions that have occurred since the divergence of this group, molecular studies in this group are important for understanding and contextualizing physiological shifts throughout vertebrate evolution.

Modern lampreys are iono- and osmoregulators, in that they maintain their plasma osmolality at approximately one-third that of seawater (SW) regardless of external salinity. This osmoregulatory strategy is found in nearly all other extant vertebrate lineages, with the notable exception of elasmobranchs, coelacanths, and lungfishes^11,12^. The persistence of an osmoregulatory system over evolutionary time underscores its adaptive importance, particularly considering the extensive increases in phenotypic complexity that has occurred in derived fishes^13^*.*

Like marine teleosts, lampreys ingest large amounts of SW, which are progressively processed by distinct intestinal regions to compensate for passive water loss^14^. The gut desalinates ingested SW until it is nearly iso-osmotic with respect to the blood^14,15^, thus facilitating net water absorption^16^. Intestinal water absorption occurs primarily via two possible paths: (i) transcellular, in which aquaporins^17^, Na^+^-glucose transporters^18,19^, and/or Na^+^-K^+^-2Cl^−^ (NKCC2)/Na^+^-Cl^−^ (NCC) cotransporter proteins are involved^20^; (ii) paracellular diffusion across the tight apical junction complexes^21,22^.

The osmotic force for water absorption from the lumen is driven by salt transport and facilitated along the gastrointestinal tract by luminal CaCO_3_ precipitation^21^. This process begins with ingested SW filling lateral intercellular spaces between ion-transporting enterocytes^23^. The intestinal water absorption from the lumen into the serosa likely occurs by osmosis following Cl^−^ molecular transport^21^. Note that most studies reporting “macro” osmotic gradients in the intestinal lumen and blood of marine fishes show no differences in osmotic pressure^24^. The mechanism for luminal Cl^−^ uptake is species- and tissue-dependent but always includes a member of the SLC family of solute-carrying proteins such as NKCC2, NCC, and/or Cl^−^/HCO_3_^−^ exchanger^25–27^. The active transport of Cl^−^ across the intestinal epithelium is associated with high levels of Na^+^/K^+^-ATPase (NKA) activity^28^. The basolateral NKA produces favorable electrochemical gradients for the absorption of Cl^−^ via the SLC cotransporters and anion exchangers localized in the apical and basolateral membranes of enterocytes^14,20,24^. Excess monovalent ions that are incorporated into the blood plasma are secreted across the gills^29^.

The NCC is perhaps most well-known for its crucial role in renal ion reabsorption in the mammalian distal convoluted tubule, where 5–10% of the filtered Na^+^ and Cl^−^ are reabsorbed^30–32^. Expression of an orthologous NCC (termed NCC1) in teleost fishes acclimated to freshwater (FW) environments has been shown to be highly expressed in the kidney, presumably fulfilling a similar role in NaCl absorption^33,34^. Fishes have another NCC gene (termed NCC2), first discovered and named by Hiroi et al.^35^, which is classically referred to as a ‘fish-specific NCC’, although it has not been found in sharks^34^. In teleost fishes, NCC2 is localized to the apical membrane of branchial ionocytes and is responsible for branchial NaCl absorption in FW^36–39^. Within the NKCC1/NKCC2/NCC gene superfamily, NCC1 and NCC2 form distinct lineages, indicating that this gene family has functionally diverged on an evolutionary timescale^40^.

In teleost fishes, NKCC2 is the predominant cation-chloride cotransporter that facilitates apical ion absorption in the SW intestine^27,28,41^. Ion and water uptake by the intestine has been shown to be inhibited by bumetanide, a selective inhibitor of NKCC^20^. The NCC2 appears to have a role in Na^+^ and Cl^−^ removal from ingested SW in the esophagus and intestine of the Japanese^42,43^ and European eel^44^. However, information regarding the importance of NCC in intestinal ion and water uptake in other fish species is still scarce^20^.

Although these underlying mechanisms for intestinal SW osmoregulation have been elucidated in teleost fishes, whether such molecular mechanisms are present in the lamprey intestine was unknown prior to the present study^28^. Thus, we aimed to functionally characterize the putative molecular pathway for intestinal ion absorption during SW osmoregulation in the sea lamprey (*Petromyzon marinus*). Sea lamprey has a complex anadromous life cycle marked by a dramatic metamorphosis from a benthic filter-feeding larva (termed ‘ammocoete’) into a parasitic juvenile that migrates from FW to SW. After ~ 2 years at sea, the adults migrate upstream to spawn. Although the larval-juvenile metamorphosis has been morphologically conserved from at least the early Cretaceous, ~ 125 mya^45^, recent studies on fossil records suggest that ammocoetes are specializations of modern lampreys rather than a relic of ancient lampreys^46^.

We hypothesized that intestinal ion absorption in SW-acclimated sea lamprey occurred by an NKCC and/or NCC. We present the discovery of two functionally divergent NCC isoforms in lamprey, which appear to be basal in structure and function to the NCC isoforms of more derived fishes. Moreover, we show that NCC and not NKCC2 is the likely pathway for ion absorption in the intestine of marine lampreys.

## Results

### Tissue profile and molecular characterization

**Figure 1:**
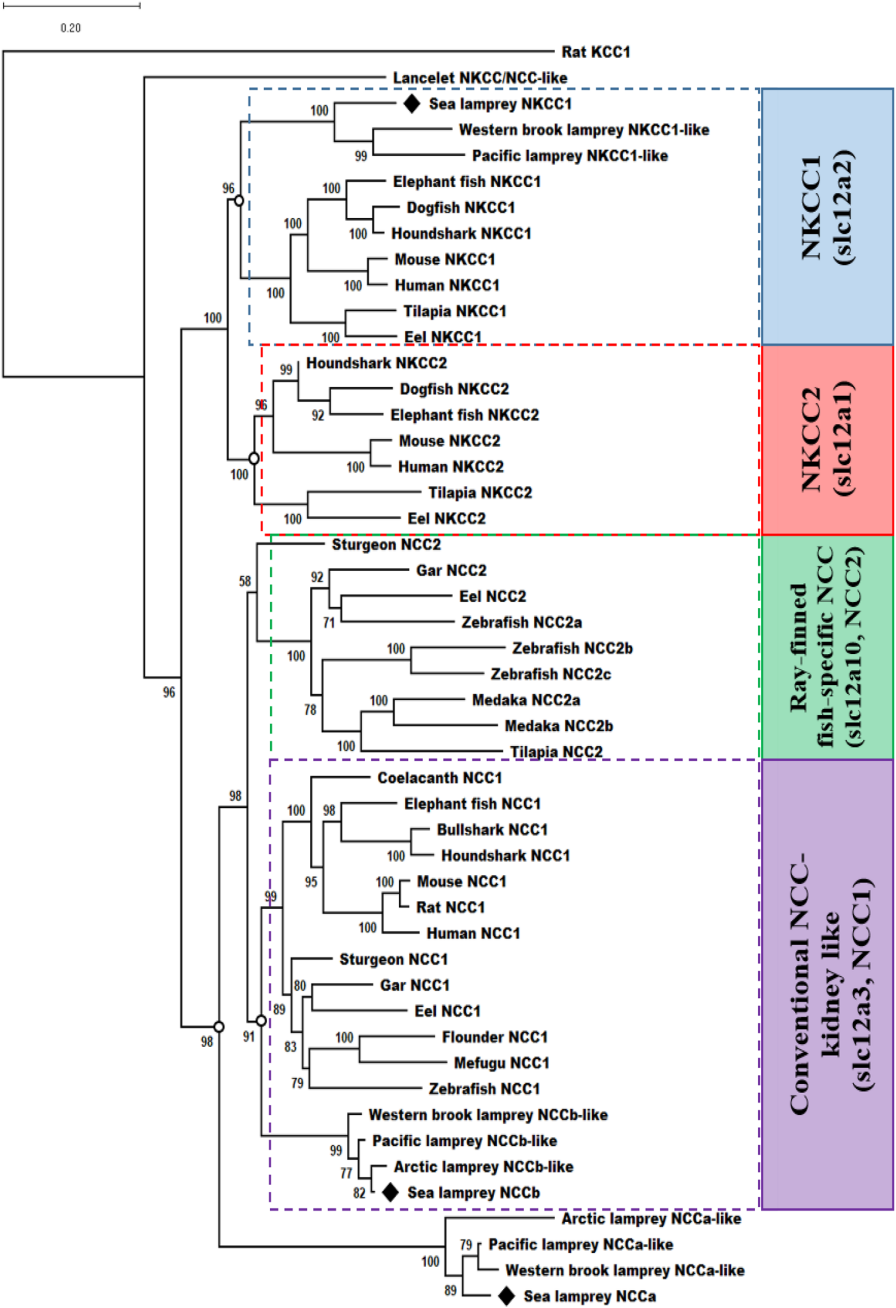
Molecular phylogenetic tree of vertebrate NKCC/NCC family. Rat KCC1 was used as an outgroup to root the tree.

We comprehensively surveyed cDNA sequences encoding relevant ion-transporting proteins in the sea lamprey genome. Partial sequences of orthologous cDNAs encoding two distinct NCC-like proteins were discovered. The accession numbers of these newly identified sea lamprey NCC sequences are BK014291 and BK014292, which we have named “NCCa” and “NCCb”, respectively. We could not identify a gene encoding an NKCC2-like protein in any available genome for different lamprey species. Deduced amino acid sequences for sea lamprey NCCa and NCCb shared the highest sequence identity to known NCC families; molecular phylogenetic analysis grouped NCCa and NCCb with other NCC sequences (Fig. 1). NCCa occupied a basal position among the NCC1 and NCC2 clades. NCCb was basally positioned within the clade of the conventional, kidney-specific NCC1 sequences.

**Figure 2:**
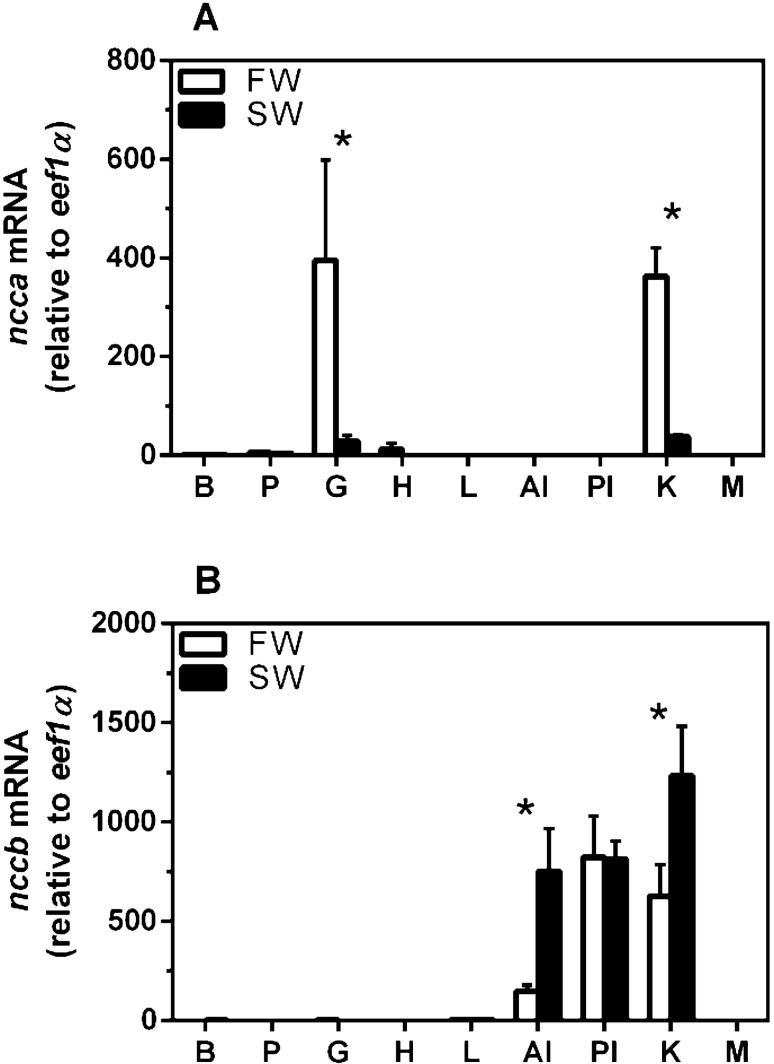
Gene expression of *ncca* (**A**) and *nccb* (**B**) isoforms in a tissue profile of juvenile sea lamprey acclimated to either FW or SW. Abbreviations: B, brain; P, pituitary; G, gill; H, heart; L, liver; AI, anterior intestine; PI, posterior intestine; K, kidney; M; muscle. Values are relative to the FW brain tissue. Each column represents mean ± SEM (n = 3). *Significant differences within a given tissue between environmental salinities (two-way ANOVA, Tukey’s post hoc).

In tissue profiles, mRNA abundance of *ncca* and *nccb* showed drastically different expression patterns. The *ncca* was expressed predominately in the gills and kidney at ~ 400-fold higher levels than other tissues in FW-acclimated juvenile sea lamprey (Fig. 2A). Acclimation to SW resulted in ~ 90% reduction of *ncca* in the gills and kidney. The *nccb* was predominately expressed in the anterior intestine (AI), posterior intestine (PI), and kidney at ~ 1000-fold higher levels than other tissues in SW-acclimated sea lamprey (Fig. 2B). The *nccb* mRNA in the AI and kidney of SW-acclimated sea lamprey was 5- and twofold higher, respectively, than in FW-acclimated sea lamprey.

**Figure 3:**
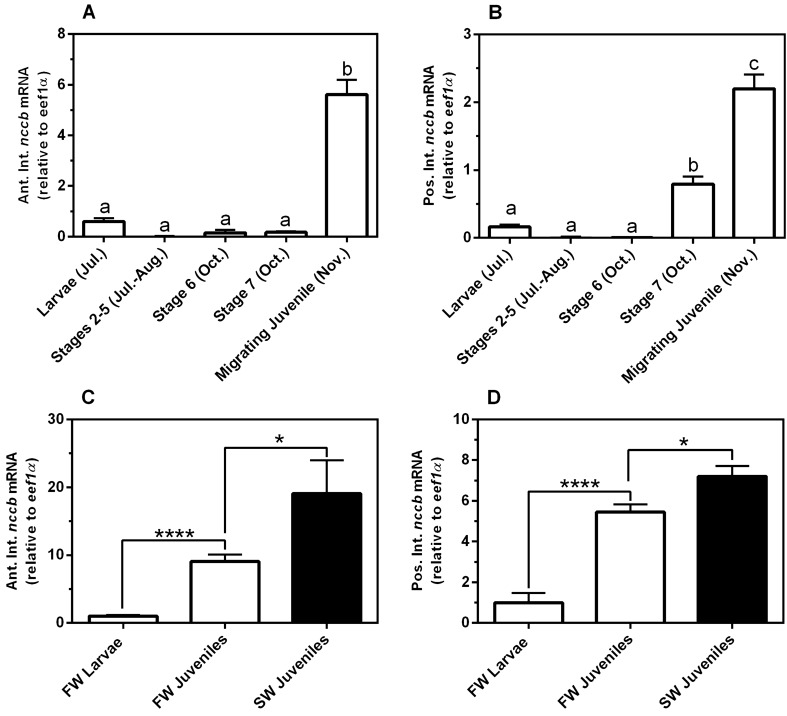
Gene expression of *nccb* in the anterior, AI (**A**) and posterior intestine, PI (**B**) during metamorphosis of the sea lamprey. Gene transcription of *nccb* in the AI (**B**) and PI (**C**) for larvae, and FW- and SW-acclimated juvenile sea lamprey. Each column represents mean ± SEM (n = 4–19). Different superscript letters indicate significant differences within intestinal regions among life stages (**A** and **B**; P < 0.05, one-way ANOVA, Tukey’s post hoc). *Significant differences within the same environmental salinities between groups (**B** and **C**; unpaired Student’s t-test).

In additional metamorphic and salinity experiments, *nccb* in the intestine increased during the late stages of metamorphosis and then increased further after SW acclimation. The *nccb* mRNA levels increased six and fivefold in the AI and PI, respectively, throughout the larvae-to-juvenile metamorphosis (Fig. 3A,B). In lab-acclimated sea lamprey, intestinal *nccb* mRNA was significantly higher in both the AI (ninefold) and PI (5.5-fold) in FW-acclimated juveniles compared to larvae (Fig. 3C,D). Acclimation to SW further increased sea lamprey *nccb* mRNA abundance in the AI (twofold) and PI (1.5-fold).

### Electrophysiological properties of sea lamprey intestine

**Figure 4:**
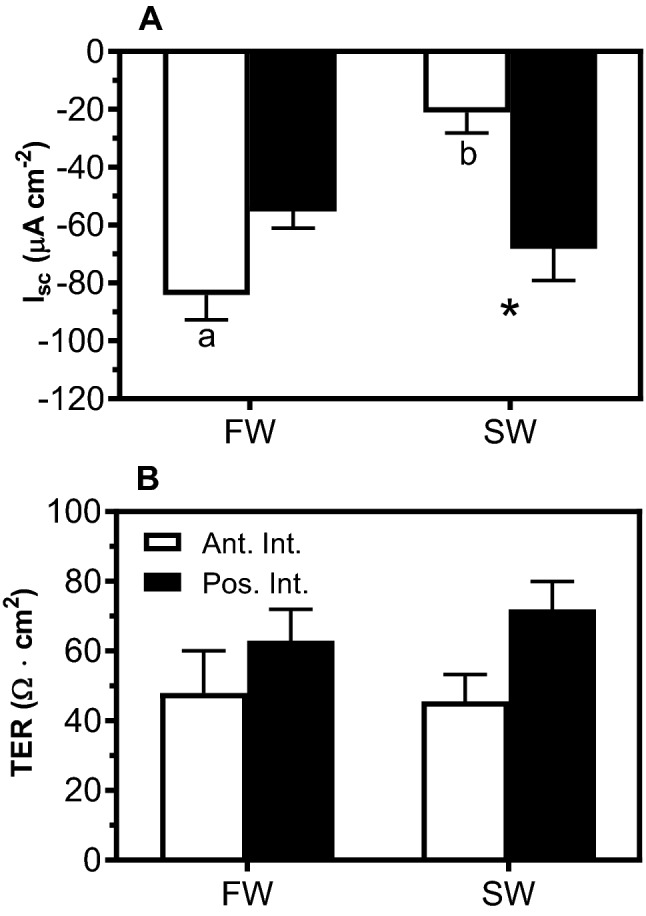
(**A**) Short-circuit current (I_sc_, μA cm^−2^), and (**B**) transepithelial electric resistance (TER, Ω·cm^2^) in the anterior (AI) and posterior (PI) intestine of FW- and SW-acclimated juvenile sea lamprey. Each column represents mean ± SEM (n = 8–15). Different letters indicate significant differences within intestinal regions between environmental salinities. *Significant differences between intestinal regions within salinities (two-way ANOVA, Tukey’s post hoc).

Briefly, we measured ‘transepithelial’ electrophysiology possibly due to coordinated fluxes across the apical and basolateral membranes. The resulting paracellular Na-diffusion flux (*i.e.*, sodium and chloride inwards and Na diffusing paracellularly outwards), a mechanism common to other NaCl absorbing epithelia, sets up a transepithelial electric potential (TEP)^47^. Therefore, no particular mechanism dictates the global TEP across the epithelium, such as electroneutral NKCC/NCC. The combined activity generates this potential, and a large TEP ex vivo is characteristic of epithelia with robust transepithelial transport activity. Chloride is the primary physiologic anion and the ion suspected of carrying the majority of the current (I_sc_). The TEP and I_sc_ values observed for FW- and SW-acclimated sea lamprey intestine are consistent with a preferential anion-absorbing tissue. In the AI, I_sc_ was significantly less negative after SW acclimation (Fig. 4A). I_sc_ of the AI of SW-acclimated sea lamprey was significantly less negative than that of the PI.

The transepithelial electric resistance (TER) did not change across intestinal regions and/or salinities (Fig. 4B).

**Figure 5:**
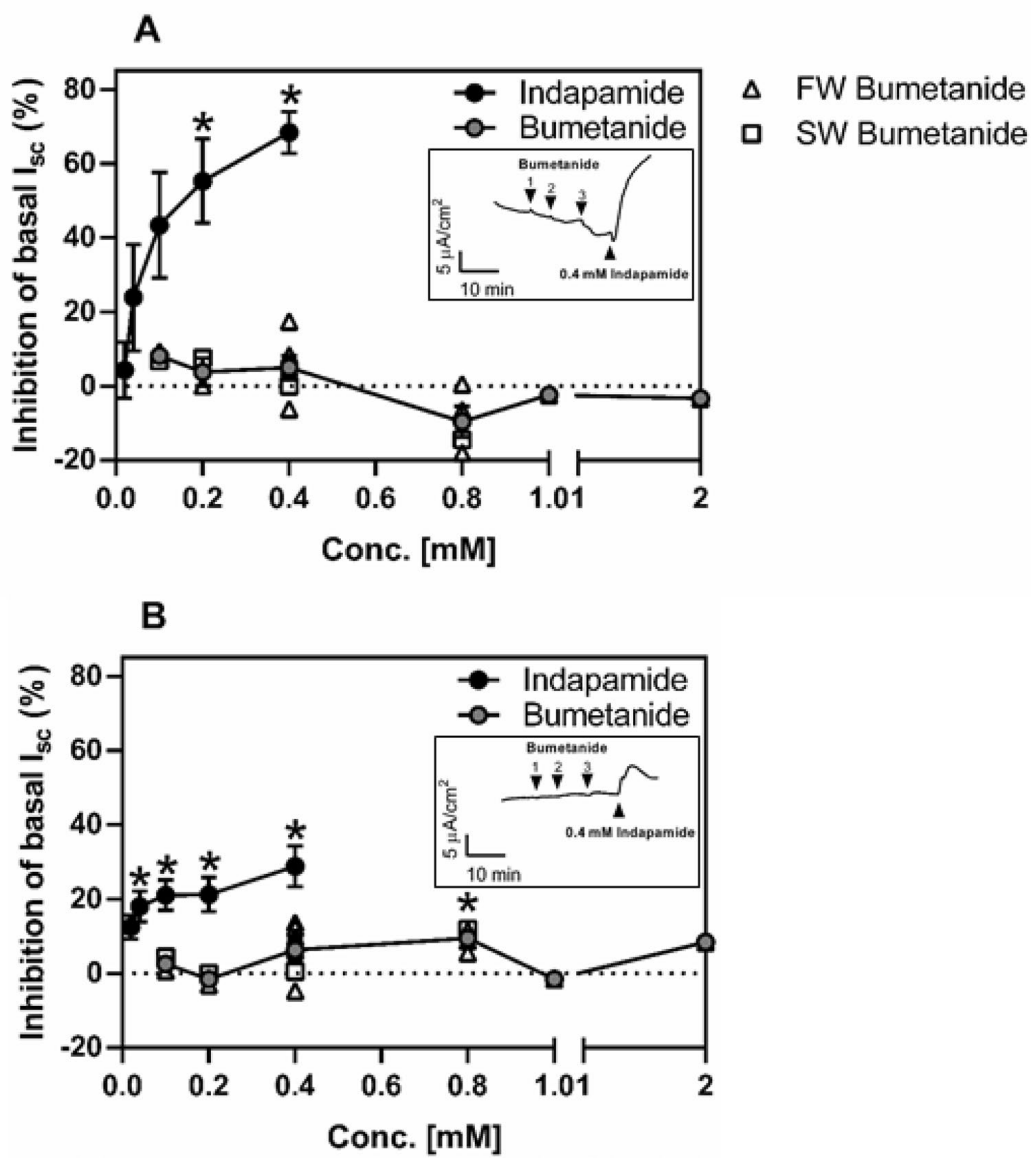
Impact of increasing concentrations of bumetanide or indapamide on basal short circuit current (I_sc_, μA cm^−2^) in the anterior (AI; **A**) or posterior (PI; **B**) intestine of juvenile sea lamprey acclimated to FW and/or SW. After mounting the tissues, the I_sc_ was allowed to stabilize (*i.e*., baseline level) before adding the respective inhibitors. Note, the grey circles represent the mean response of intestinal tissues of FW-acclimated (open triangles) and SW-acclimated (open squares) sea lamprey at each concentration of bumetanide tested. In contrast, the effect of increasing concentrations of indapamide were only examined in intestinal tissues of SW-acclimated sea lamprey. The inset figures are examples of the impact on the I_sc_ of a single application of indapamide (0.4 mM) following the last 0.4 mM bumetanide application. Data are the mean ± SEM (n = 2–6). *Significant differences between the basal I_sc_ and the I_sc_ after it reached its maximal change following drug application at each concentration of drug tested (one-sample t-test).

To investigate whether NKCC2 and/or NCCb are involved in intestinal ion absorption mechanisms in the intestine of the juvenile sea lamprey, increasing concentrations of either bumetanide in preparations from FW- and SW-acclimated juveniles, or indapamide in preparations from SW-acclimated juveniles, were added to the luminal side of the AI (Fig. 5A) and PI (Fig. 5B). Note that indapamide was employed just in SW-acclimated juveniles since their sensitivity was more remarkable than FW-acclimated ones (data not shown). There was no significant effect of bumetanide at any of the concentrations tested (0.1–2.0 mM). In contrast, increasing concentrations of indapamide resulted in significant inhibition of the baseline I_sc_, and the effect appeared to be dose-dependent. The inset figures are original traces of the impact on the I_sc_ of a single application of indapamide (0.4 mM) following the last application of 0.4 mM bumetanide. These data are consistent with the presence of an NCC-mediated anion absorptive pathway in both the AI and PI regions of the SW-acclimated sea lamprey intestinal tract.

**Figure 6:**
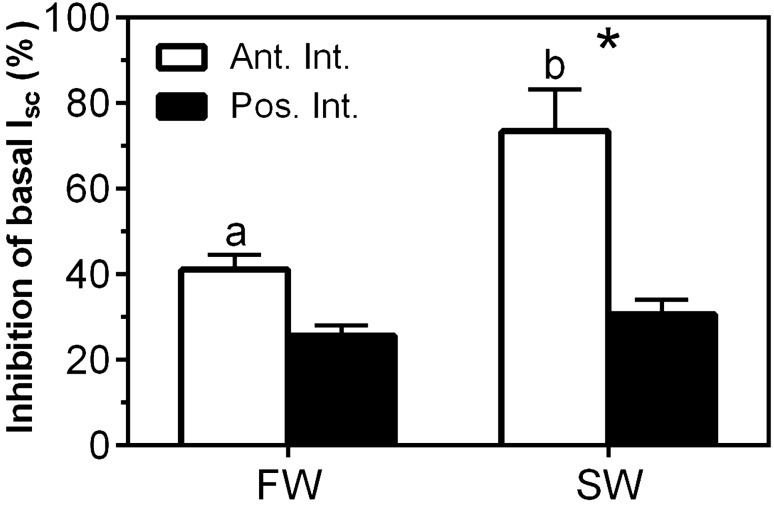
Percent inhibition of baseline short circuit current (%) in response to luminal indapamide (0.4 mM) in FW- and SW-acclimated juvenile sea lamprey. All data are the mean ± SEM (n = 5–8). Different letters indicate significant differences within intestinal regions between environmental salinities. *Significant differences between intestinal regions within environmental salinities (two-way ANOVA, Tukey’s post hoc).

The magnitude of the indapamide effects varied between intestinal regions and with environmental salinity (Fig. 6). In FW, approximately 40% and 25% of the baseline anion absorption was indapamide-sensitive in the AI and PI, respectively. In SW, indapamide-sensitive anion absorption was significantly higher in the AI than in the PI. Indapamide-sensitive anion absorption in the AI was 73% of baseline anion absorption in SW-acclimated juvenile sea lamprey, compared to only 30% of that PI.

### Intestinal water absorption

**Figure 7:**
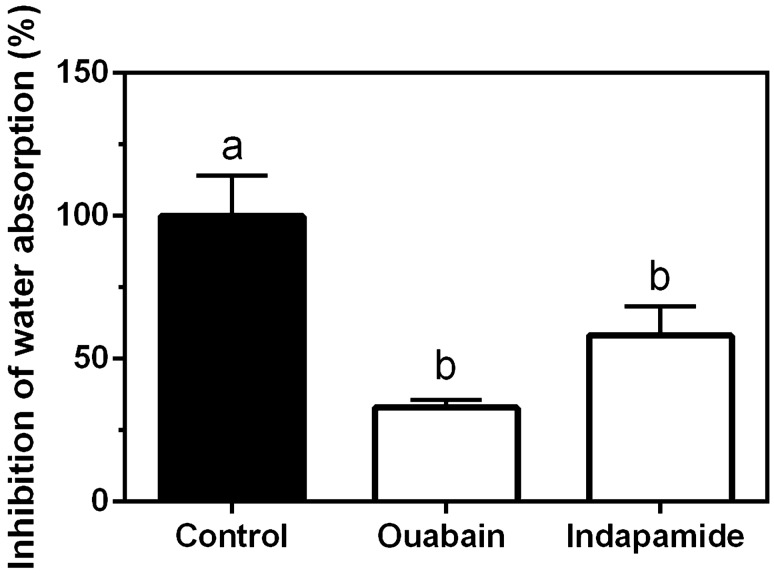
Intestinal water absorption (% of control) in whole intestines of juvenile sea lamprey acclimated to SW. Ouabain (500 µM) was added to the serosal side, and indapamide (400 µM) was added to the luminal side. All data are presented as mean ± SEM (n = 3–4). Different letters indicate significant differences among groups (one-way ANOVA, Tukey’s post hoc).

Whole intestine water absorption under iso-osmotic conditions decreased significantly by 64% and 41% after the serosal addition of ouabain (500 µM) or luminal addition of indapamide (400 µM), respectively (Fig. 7).

## Discussion

It has been hypothesized that a first-round of whole-genome duplication (1R) in vertebrates led to an initial divergence of NKCC from NCC and that a second round of whole-genome duplication (2R) in the vertebrate lineage resulted in multiple NKCC and NCC isoforms, NKCC1/NKCC2 and NCC1/NCC2^48^. Although Agnathans radiated after 1R or 2R is still unresolved^6,49^, recent studies in *noggin* genes in lampreys suggest two rounds (2R) of ancient genome duplication^50^.

Our molecular phylogenetic analysis showed that the NCC1 and NCC2 of bony fishes were grouped into distinct NCC1 (conventional, kidney NCC) and NCC2 (ray-finned fish-specific gill NCC) clades. Sea lamprey NCCb was clearly positioned at a basal position within the NCC1 clade. Sea lamprey NCCa was not explicitly located within either the NCC1 or NCC2 clades and was instead positioned basal to both NCC clades. All retrieved NKCC1-, NCCa-, and NCCb-like proteins from other lamprey species were grouped with the respective identified sequences of the sea lamprey (*Petromyzon marinus*).

The dramatic changes in *nccb* transcription during the latest stages of development and in response to environmental changes are good indicators of functional relevance. We found that *nccb* mRNA levels were significantly upregulated in the later stages of metamorphosis, particularly in post-metamorphic juveniles which were captured while actively migrating toward the sea. Barany et al.^14,51^ observed increases in intestinal NKA transcription, mRNA, and activity in the late stages of metamorphosis. Both NKA and NCCb might be important to the development of SW tolerance that occurs late in metamorphosis. Based on our transcriptional and pharmacological experiments, NCCb in the intestine was further upregulated in juveniles after exposure to SW. Furthermore, the expression and activity of NCCb in SW were higher in AI than in PI. These changes in intestinal NCCb expression and activity during metamorphosis and SW-acclimation reflect similar intestinal NKA activity and expression^14^, indicating that both of these transporters play a critical role in developing salinity tolerance in lamprey. This salinity-responsive pattern for NCCb is similar to reports on the apical NKCC2 in the intestine of euryhaline teleosts transferred to higher salinities^27,52,53^, and on the basolateral NKCC1 in the gills of sea lamprey^54^. The SW adaptive changes in gill NKCC1 expression have been shown to be driven by corticosteroid signaling^55^. It has also been shown that the intestine of post-metamorphic juvenile sea lamprey highly expresses a corticosteroid receptor^51,56^. Thus, whether SW-adaptive anion absorption via NCCb in the AI is also under corticosteroid control should be investigated.

Although the present study was explicitly focused on functional characterization of the intestinal NCCb isoform, we posit a potential mechanism for branchial ion uptake and renal NaCl reabsorption in FW-acclimated sea lamprey, the NCCa isoform. In our mRNA tissue profile, *ncca* was highly expressed in the gills of FW-acclimated juvenile lamprey and significantly down-regulated after SW acclimation. This apparent FW-specific role for *ncca* in the gills may explain the apparent FW-specific regulation of an *ncc*-like orthologue reported in the gills of up-stream migrating adult sea lamprey^57^. This salinity-specific regulation of gill NCC in order to adapt to different salinities is similar to what has been described of gill NCC2 in teleosts^35,39,58^.

The expression of both NCCs, NCCa and NCCb, in the sea lamprey kidney, is unique among vertebrates and warrants further investigation. The sea lamprey *ncca* was abundant in the kidney of FW-acclimated juveniles and strongly down-regulated after SW acclimation. By contrast, *nccb* mRNA levels in the kidney were upregulated after SW acclimation. The typical salinity-responsive pattern of the conventional kidney NCC1 in fishes is that it is abundant in FW and down-regulated in SW^33^. This indicates that NCCa in sea lamprey kidney functions similar to the conventional, kidney-specific NCC involved salt retention of teleosts in FW. The expression of the sea lamprey *ncca* in both the gills and kidney of FW-acclimated sea lamprey is particularly interesting because, in teleost fishes, the NCC in the gills (NCC2) and kidney (NCC1) are two distinct NCC isoforms^33,35,37^. It is important to note that the elasmobranch NCC also exhibits FW-specific function in the gills and kidney^34,59^, similar to the expression pattern of the sea lamprey NCCa.

In our effort to functionally characterize the intestinal NCCb isoform, we used an electrophysiological approach to elucidate the underlying mechanisms contributing to intestinal ion transport in response to environmental salinity. The anion-absorptive properties of the sea lamprey intestine reported here agree with what has previously been shown in the sea lamprey intestine^14^. Surprisingly, absorptive I_sc_ in the AI was higher in FW-acclimated juvenile sea lamprey. It is known that FW-acclimated sea lamprey exhibits some minimal drinking^14,60^. Thus, it could be that the ion uptake capacity of the FW-acclimated AI offers extra osmoregulatory support by facilitating ion uptake in ion-poor environments, suggesting that intestinal ion uptake from imbibed FW might be more important than previously thought. In this case, the AI would supplement branchial uptake in FW. Although intestinal ion uptake from feed could supplement ion uptake by the gill in freshwater larvae, feeding by migrating anadromous juveniles does not usually occur until fish reaches seawater. In SW, higher anion-absorptive I_sc_ was detected in the PI than in the AI. This demonstrates that these intestinal regions have different functional responses to environmental salinity, similar to what has also been previously reported in teleosts^27,41,61^. We anecdotally observed intestinal precipitates only in the intestine of SW-acclimated juveniles (data not shown). Thus, the reduction in anion absorption in the AI of SW-acclimated juveniles may also indicate a functional switch in intestinal physiology to secrete HCO_3_^−^ and/or H^+^^62^ into the AI lumen to lower luminal osmolality and facilitate net water absorption^63,64^. Although no direct reports of measurements of intestinal fluid HCO_3_^−^ concentrations or secretion exists for lamprey, reports by Rankin^65^ show a substantial cation–anion change difference among measured ions in the intestinal lumen of SW-acclimated lamprey. Moreover, this observation and what it may imply for evolutionary aspects of intestinal HCO_3_^−^ secretion by marine fishes was discussed by Taylor and Grosell^66^. Still, further research is needed to determine if a similar gut intestinal fluid alkalinization mechanism occurs in sea lamprey.

In the juvenile sea lamprey, the apparent lack of sensitivity to bumetanide indicates that anion absorption by NKCC2 is unlikely to be present in the sea lamprey intestine. This is further supported by our inability to identify a gene encoding NKCC2 in the published genomes of sea lamprey, western brook lamprey, Pacific lamprey, or Arctic lamprey. We cannot rule out the possibility that a sea lamprey NKCC2 could be insensitive to bumetanide^67,68^ and that *nkcc2* is present but has yet to be detected in the available lamprey genomes. Note that no significant effects in I_sc_ were obtained with either chlorothiazide or hydrochlorothiazide, which were initially employed to target a putative NCC. It also should be noted that NKCC1 has been identified and characterized in sea lamprey gills^54^ but is present only at very low levels in the intestine. Moreover, we were able to identify NKCC1-, NCCa-, and NKCCb-like proteins in the available different lamprey species genomes. Therefore, we can surmise two possible explanations for the lack of a sea lamprey *nkcc2*: (i) Agnathans diverged after 2R, but without a functional role, an *nkcc2* gene was eventually lost; and (ii) Agnathans diverged before 2R, and an *nkcc2* gene never existed in sea lamprey.

Our results demonstrate that an indapamide-sensitive mechanism (*i.e.,* NCCb) on the apical membrane appears to be a major contributor to anion absorption in the lamprey intestine. Overall lumen-to-serosa ion influx along the intestinal tract was observed, with marked differences depending on intestinal regions. In SW-acclimated sea lamprey juveniles, approximately 70 and 30% of the net absorptive currents in the AI and PI, respectively, are mediated by NCCb. With NKCC2 ruled out by the lack of bumetanide sensitivity, any further speculation about the mechanisms facilitating the residual I_sc_ that is NCCb-independent (*i.e.,* insensitive to indapamide) is beyond the scope of this investigation. Further studies are required to elucidate whether the NKCC2/NCC-independent mechanisms of Cl^−^ absorption, which have been shown in the teleost intestine^28^, are also present in lampreys. It is also possible that indapamide simply did not fully inhibit all NCCb activity, even at the highest tested doses. Further pharmacological characterization of the sea lamprey NCCs is warranted.

The contribution of NCCb to anion-absorption in the AI is about twice as high in SW compared to FW. Thus, increased environmental salinity may cause a shift of Cl^−^ absorption mechanisms^41^, which agrees with previously reported increases in drinking rate, intestinal NKA activity, and intestinal water absorption^14^, as well as increased gill Cl^−^ secretory mechanisms^54^. The higher sensitivity of I_sc_ to indapamide in the AI than the PI is consistent with higher *nccb* transcription in this region.

To better understand how intestinal water absorption was related to NCCb activity, we characterized indapamide-sensitive intestinal water transport in SW-acclimated sea lamprey juveniles. As expected, the serosal addition of ouabain inhibited active water absorption, demonstrating NKA is critical involvement in this process, as has previously been shown in teleosts and sea lamprey^14,69^. The luminal addition of indapamide also inhibited water absorption to a similar degree as the addition of ouabain. These results indicate that the activity of NCCb in the sea lamprey intestine is a critical ion absorptive mechanism that is functionally coupled to water absorption.

In summary, our finding that an NCCb, and not NKCC2, functions in the intestine offers new insights into the diversification of the vertebrate NKCC/NCC protein superfamily as it might be related to partial and/or whole-genome duplication events. Although the results reported here are an important discovery and describe specific differences in the osmoregulatory roles of NKCCs and NCCs between lamprey and later-derived fishes, further studies are still needed to complete our understanding of ion-absorptive pathways in the gill, kidney, and intestine of the sea lamprey.

## Materials and methods

### Animals and experimental designs

All experiments were carried out in accordance with USGS guidelines and approved by the USGS Institutional Animal Care and Use Committee (Protocol No. LB00A3O-117). In addition, the study reported here also is in accordance with ARRIVE guidelines. Sea lamprey juveniles were caught in the Sawmill River, a tributary of the Connecticut River (Montague, MA, USA), by electrofishing (ammocoetes and stages 1–7) or Fyke net capture (considered ‘migrating juveniles’) from July to November 2018. For the metamorphic series, lamprey were sampled in the field immediately upon capture and staged according to the descriptions presented by Youson et al.^70^. Salinity acclimation experiments were carried out under laboratory conditions at the USGS Conte Anadromous Fish Research Center (Turners Falls, MA, USA) and acclimated to experimental salinity conditions for least 3 weeks prior to sampling. Experimental SW was prepared by mixing artificial sea salt (Crystal Sea Salt, Baltimore, MD, USA) and dechlorinated municipal FW. Lamprey in FW and SW were kept under natural photoperiod conditions and at a constant temperature of 15 ºC in 60 L recirculating glass aquaria equipped with mechanical, chemical, and biological filtration and monitored daily. The animals were not offered food because they naturally stop feeding during metamorphosis and do not resume until they begin parasitic feeding once they reach the ocean.

### Sampling protocol

For all tissue collection, lamprey were euthanized using MS-222 (400 mg L^−1^ buffered with NaHCO_3_, pH 7.0) (Argent Chemical Laboratories, Redmond, WA, USA). Tissue was collected from the gills and two sections of the intestine: (i) the AI, corresponding to 1.5–2 cm after the end of the esophagus; and (ii) the PI, which corresponds to a section of distal intestine, 2–3 cm in length, delimited by the rectal sphincter. All samples were immediately frozen and stored at − 80 °C until analysis, except in cases of measurements of intestinal water permeability and electrophysiology, where the tissues were used immediately after dissection.

### Gene identification and molecular phylogenetic analysis

Gene-specific primers were designed based on sea lamprey genomic information available from the SIMRbase (https://genomes.stowers.org/). The identity of annotated genes was confirmed using sequence identity comparison and NCBI blast analyses.

The deduced amino acid sequences of the obtained annotated genes were predicted using the Translate tool provided by the Expasy bioinformatics resource portal (https://www.expasy.org). Phylogenetic analysis was carried out on a balanced selection of vertebrate NKCC1, NKCC2 and NCC peptides available from NCBI GenBank, Ensemble Genomes and SIMRbase, using a ClustalW alignment (https://www.ebi.ac.uk/clustalw) and the neighbor-joining method with bootstrap analysis for 1000 cycles implemented by MEGA X software. Accession numbers for the peptides used in the phylogenetic analysis were: lancelet (NKCC/NCC-like, XP_035666237.1), human (NKCC1, U30246; NKCC2, NM_000338; NCC1, X91220), mouse (NKCC1, NP_033220.2; NKCC2, NP_899197.2; NCC1, NP_062288.1), rat (NCC1, NP_062218.3; KCC1, NM_019229.2), tilapia (NKCC1, AY513737; NKCC2, AY513739; NCC2, EU518934), eel (NKCC1, AJ486858; NKCC2, AJ564602; NCC1, AJ564604; NCC2, AJ564606), zebrafish (NCC1, NP_001038545; NCC2a, NP_001154850; NCC2b, EF591989; NCC2c, NP_001128603.1), medaka (NCC2a, KJ489428; NCC2b, KJ489429), mefugu (NCC1, AB479211), flounder (NCC1, L11615), gar (NCC1, ENSLOCG00000007841; NCC2, ENSLOCG00000000965), sturgeon (NCC1, XP_033895975.2; NCC2, XP_034770356.1), coelacanth (NCC1, XM_014494272), elephant fish (NKCC1, AB769492; NKCC2, AB769493; NCC1, AB769494), dogfish (NKCC1, U05958; NKCC2a, AF521915), bullshark (NCC1, AB769491), houndshark (NKCC1, AB669487; NKCC2, AB769486; NCC1, AB769487), arctic lamprey (NCCa-like, KE993714.1; NCCb-like, APJL01019054.1); western brook lamprey (NKCC1-like, LPT_00008897-P; NCCa-like, LPT_00002495-RA; NCCb-like, LPT_00012662-RA); pacific lamprey (NKCC1-like, ETRf_mk00026850-P; NCCa-like, ETRf_mk00025483-RA; NCCb-like, ETRf_mk00025494-RA); sea lamprey (NKCC1, MK779970.1; NCCa, BK014291; NCCb, BK014292).

### RNA isolation and quantitative real-time PCR

Total RNA was extracted from frozen tissues using TRIzol reagent (Molecular Research Center Inc., Cincinnati, OH, USA) following the manufacturer’s protocol. The total RNA concentration and purity of each sample were determined spectrophotometrically using a Take3 microvolume plate reader (BioTek Instruments, Inc., Winooski, VT, USA), and RNA integrity was assessed using gel electrophoresis. Only high-purity RNA (1.9 < A260/A280 > 2.2) were used for cDNA synthesis. First-strand cDNA synthesis was accomplished using a High-Capacity Reverse Transcription Kit following the manufacturer’s protocol (Applied Biosystems, Carlsbad, CA, USA). Quantitative real-time PCR (RT-qPCR) was carried out in 10 µL reactions containing 5 ng cDNA template (estimated from the input of total RNA), 150 nM forward and reverse primers, and SYBR Select master mix (ThermoFisher, Waltham, MA, USA). All RT-qPCR reactions were performed in optical 96-well reaction plates in a StepOnePlus™ Real-Time PCR System (Applied Biosystems, Inc., Foster City, CA, USA) covered with adhesive seals. The thermocycling procedure was as follows: an initial step of 50 °C (2 min) followed by 95 °C (2 min), and then 40 consecutive cycles of 95 °C (15 s), 60 °C (1 min), and 72 °C (30 s). Melting curves were used to ensure that only a single PCR product was amplified and to verify the absence of primer-dimer artifacts. A pool of concentrated cDNA from all samples for each tissue was used for a standard curve, which was a five-point, serial tenfold dilution from 50 ng to 5 pg. The linearity and efficiency of amplification for each primer combination was assessed. Control reactions with RNase-free water (no template control) and RNA (no reverse transcriptase during cDNA synthesis) were included in the analysis to ensure the absence of primer-dimerization and genomic DNA contamination. For all pairs of primers, linearities (*R*^2^) and amplification efficiencies were in the ranges 0.998–1 and 0.923–0.939, respectively. The relative gene expressions were quantified using the ΔΔC_T_ method^71^, corrected for efficiencies^72^, and referenced to the relative mRNA expression of the difference between the target gene and an endogenous reference gene (*eef1α;* gene encoding eukaryotic elongation factor 1-alpha) for each sample. Primer sequence details are shown in Table 1.

**Table 1:** Primers used for real-time qPCR expression analysis.

Gene name	Nucleotide sequence (5′→3′)		GenBank acc. no	Amplicon size (bp)
*ncca* (^a^)	Fw	GTCATCACGGTCACCTTCCT	BK014291	289
	Rv	ACACCGGAGTGAAATTCTCG		
*nccb*	Fw	GATCCTGCTGGACTACTCGC	BK014292	177
	Rv	CAGTACAGGGTGAGCACGTT		
*eef1α*	Fw	GTGGGTCGTGTTGAGACTGG	KU726618	208
	Rv	GGTCGTTCTTGCTGTCAC		

GenBank accession number referred to the unique sequence identifier in the gene assembly and sizes of the amplified products (bp).

^a^Primers from^57^.

### Chemicals

Bumetanide (a selective NKCC2 inhibitor), ouabain (a selective NKA inhibitor), and indapamide (a selective NCC inhibitor) were purchased from ThermoFisher (Waltham, MA, USA) and prepared as concentrated stocks in dimethyl sulfoxide (DMSO) except ouabain, which was dissolved in lamprey serosal saline. The final volume of DMSO never exceeded 0.2%, and no effect of DMSO at this concentration was observed on multiple tissues tested (data not shown).

### Electrophysiology in ussing chambers

Anterior and posterior intestinal tissues were dissected, washed, and mounted in Ussing chambers. The tissues were opened longitudinally, flattened, and placed between the chambers containing 1.2 mL of serosal saline (in mM: 128.0 NaCl, 1.2 NaH_2_PO_4_, 5.0 NaHCO_3_, 4.0 KCl, 2.4 CaCl_2_, 0.9 MgSO_4_, 0.9 MgCl_2_, 5.5 glucose; 270 mOsm/kgH_2_O; pH 7.8). During the experiments, the tissue was bilaterally gassed with humidified 0.5% CO_2_ in oxygen, and the temperature was maintained at 15 °C. The TEP (in mV) was referenced to the luminal side, where a positive TEP indicates that the luminal side is positively charged with respect to the serosal side. The I_sc_ (in μA cm^−2^) was monitored by clamping epithelia to 0 mV expressed as negative for the absorption of anions. The TER (in Ω·cm^2^) was manually calculated (Ohm's Law) using the current deflections induced by a 10 mV pulse at the beginning and the end of the experiment. After the tissue achieved a steady state, which usually occurred between 30 and 40 min after mounting, all data were digitally recorded using the Lab-Trax-4 acquisition system (World Precision Instruments, Sarasota, FL, USA) and LabScribe3 software (iWorx Systems Inc., Dover, NH, USA). Ag–AgCl electrodes were used as short‐circuiting electrodes to measure transepithelial electrical properties. The electrodes were connected to the serosal and luminal sides using PE90 tubing containing solidified 3 mM KCl and 2% agar. A high impedance automatic dual voltage clamp (EVC 4000‐4, World Precision Instruments, Sarasota, FL) was used for voltage clamping and monitoring TEP. Under these conditions, the measured I_sc_ is a function of transcellular ion transport that requires an energy input (active transport).

### Determination of iso-osmotic in vitro intestinal water absorption

Water absorption was determined following previous methods^14^. Briefly, the intestinal sections were isolated, flushed and then incubated for 30 min in lamprey serosal saline bubbled with a physiological gas mixture (humidified 0.5% CO_2_ in oxygen). Saline was maintained at 15 °C and pre-gassed for at least 30 min before experimentation. The ends of the intestinal samples were sealed at each end using dental line and placed in a beaker containing serosal saline supplied with the gas mixture. The intestinal preparations were weighed every 15 min for 1 h. At the end of the experiment, each intestinal section was opened, placed on graph paper, and then photographed. The surface area of the intestinal segment within the tied region was determined using ImageJ software (NIH, Bethesda, MD).

### Statistical analysis

Statistical comparisons across experimental procedures were performed using unpaired Student’s t-test, one-sample t-test, one-way ANOVA, and two-way ANOVA, as indicated in the figure legends. All data are represented as the mean ± SEM. Prior to these statistical analyses, both normality and equal variance were confirmed. When ANOVA yielded significant differences, Tukey's post hoc test was used to identify these. Statistical significance was accepted at P < 0.05.

## Data Availability

All data generated or analyzed during this study are available in Science Data Bank with the following https://doi.org/10.11922/sciencedb.01279.
